# Mungbean *DIRIGENT* Gene Subfamilies and Their Expression Profiles Under Salt and Drought Stresses

**DOI:** 10.3389/fgene.2021.658148

**Published:** 2021-09-22

**Authors:** Wenying Xu, Tong Liu, Huiying Zhang, Hong Zhu

**Affiliations:** ^1^ College of Life Sciences, Qingdao Agricultural University, Qingdao, China; ^2^ College of Agronomy, Qingdao Agricultural University, Qingdao, China

**Keywords:** mungbean, VrDIR, gene expression, salt stress, drought stress

## Abstract

*DIRIGENT* (*DIR*) genes are key players in environmental stress responses that have been identified in many vascular plant species. However, few studies have examined the *VrDIR* genes in mungbean. In this study, we characterized 37 *VrDIR* genes in mungbean using a genome-wide identification method. *VrDIRs* were distributed on seven of the 11 mungbean chromosomes, and chromosome three contained the most *VrDIR* genes, with seven members. Thirty-two of the 37 *VrDIRs* contained a typical *DIR* gene structure, with one exon; the conserved DIR domain (i.e., Pfam domain) occupied most of the protein in 33 of the 37 *VrDIRs*. The gene structures of *VrDIR* genes were analyzed, and a total of 19 distinct motifs were detected. *VrDIR* genes were classified into five groups based on their phylogenetic relationships, and 13 duplicated gene pairs were identified. In addition, a total of 92 *cis*-acting elements were detected in all 37 *VrDIR* promoter regions, and *VrDIR* genes contained different numbers and types of *cis*-acting elements. As a result, *VrDIR* genes showed distinct expression patterns in different tissues and in response to salt and drought stress.

## Introduction

Mungbean is an important legume crop that is mainly grown in Asian countries, including India, Thailand, and China (Tomooka et al., 2002). Mungbean is thought to have been domesticated in India and then spread to other countries (Fuller, 2007). Mungbean provides humans with several benefits. First, the roots of mungbean can fix atmospheric nitrogen and thus improve soil fertility and texture (Graham and Vance, 2003). Second, mungbean seeds contain high amounts of protein and nutrients; for this reason, they are widely consumed (Keatinge et al., 2011). The nutritional value of mungbean increases during seed germination because of the degradation of proteins, vitamins, and minerals (El-Adawy et al., 2003). Thus, mungbean sprouts are a favored vegetable in many countries (Tomooka et al., 2002). With the rapid growth of the human population, the demand for mungbean has increased. However, the production of mungbean is affected by many environmental factors, such as salt and drought stress. The lack of knowledge of functional genes in mungbean limits our ability to enhance the resistance of mungbean to adverse environments. Generally, the study of the functions of genes in mungbean provides useful information for improving mungbean plants.

Many genes have been identified to be involved in abiotic and biotic stress, such as *DIRIGENT* (*DIR*) genes (Ralph et al., 2007; Guo et al., 2012; Paniagua et al., 2017; Khan et al., 2018; Li et al., 2019; Vaahtera et al., 2019; Han et al., 2020; Hu et al., 2020; Yu et al., 2020; Zhu et al., 2020; Liu et al., 2021). The first *DIR* reported stipulates stereoselective coupling of two coniferyl alcohol to produce (+) -pinoresinol, provided a one electron oxidase or oxidant is present. It was first identified in *Forsythia intermedia* (Davin et al., 1997); and then was later reported in *Arabidopsis* (Kim et al., 2002; Kim et al., 2012), *Schizandra chinensis* (Kim et al., 2012), *Pisum sativum* (Seneviratne et al., 2015), and *Linum usitatissimum* (Dalisay et al., 2015). *DIR* genes typically contain an exon without an intron, and the conserved DIR domain occupies most of the DIR protein (Corbin et al., 2018). *DIR* genes are found in almost all vascular plants, including ferns, gymnosperms, and angiosperms (Davin and Lewis, 2000; Ralph et al., 2007; Wu et al., 2009; Li et al., 2014). *DIR* genes have been studied at the whole genome level in many plant species, including *Arabidopsis* (25 *AtDIRs*), rice (54 *OsDIRs*), pepper (24 *CaDIRs*), *Medicago* (45 *MtDIRs*), *Brassica rapa* (29 *BrDIRs*), and soybean (54 *GmDIRs*) (Ralph et al., 2007; Guo et al., 2012; Thamil Arasan et al., 2013; Khan et al., 2018; Song and Peng, 2019; Ma et al., 2021). *DIR* genes can be classified into seven groups, designated as DIR-a to DIR-g, based on their evolutionary relationships (Ralph et al., 2006).

The functions of many DIR proteins have been identified. Members of DIR-a subfamily participate in the formation of pinoresinol (Ralph et al., 2006; Corbin et al., 2018). Several DIR-b/d subfamily members are involved in aromatic diterpenoid biosynthesis (Liu et al., 2008; Effenberger et al., 2015) and pterocarpan biosynthesis (Uchida et al., 2017; Meng et al., 2020), and the DIR-e subfamily is thought to participate in Casparian band lignin formation (Hosmani et al., 2013). In addition, many *DIR* genes have been shown to be involved in biotic stress in plants. For example, the expression of *GmDIR22* is induced by *Phytophthora sojae* infection, and the overexpression of *GmDIR22* enhances the resistance of susceptible soybean cultivar ‘Dongnong 50’ to *P. sojae* by increasing total lignan accumulation (Li et al., 2017). The expression of many *DIR* genes in spruce (*Picea* spp) was induced in response to insect attacks (Ralph et al., 2007). Many *DIR* genes also participate in abiotic stress responses. For example, the expression of *ScDIR* in sugarcane increases in response to H_2_O_2_, PEG, or NaCl stress (Guo et al., 2012). *BhDIR1* transcripts accumulate in response to changes in water and temperature stress (Wu et al., 2009). Loss of function of *CaDIR7* reduced root activity after salt stress, and the induction of stress-related genes was suppressed in *CaDIR7*-silenced plants (Khan et al., 2018). Several *BrDIR* members showed altered expression levels in response to water, ABA, and cold stress, and the expression of many *BrDIR* genes is correlated with increased lignification under water stress (Arasan et al*.*, 2013).

The study of mungbean *VrDIR* genes could provide important information for the molecular breeding of mungbean plants. The release of the mungbean draft genome sequence provides essential information for the analysis of *VrDIR* genes (Kang et al., 2014). In this study, we characterized mungbean *VrDIR* genes using genome-wide identification and investigated their phylogenetic relationships, gene structures, conserved motifs, gene duplications, *cis*-acting elements in promoters, and expression profiles in different tissues in response to salt and drought stress. Our study provides key insight into the function of mungbean *VrDIR* genes in the regulation of abiotic stress.

## Materials and Methods

### Plant Materials and Growth Conditions

The sequenced mungbean variety VC 1973A was used in all experiments in this study (Kang et al., 2014). Eight tissues, including flowers, pods, leaves, seeds, nodule roots, stems, roots, and shoot apices, were collected from mungbean plants grown in the field as described by Shi et al. (Liu et al., 2021; Shi et al., 2021). Mungbean seedlings were grown in a growth chamber for stress treatment. The growth conditions were as follows: 10 h, 28°C, light/14 h, 25°C dark cycles (light source: white fluorescent lights, ∼100 μmol m^−2^ s^−1^), and the humidity was maintained at approximately 30%. Seven-day-old mungbean plants were used for stress treatment. For salt stress treatment, plants were watered with 150 mmol NaCl, and tissues were sampled 9 days after treatment (Zhang et al., 2019; Zhu et al., 2019). For drought stress treatment, 7 day-old plants were watered, and then the plants were grown without irrigation; the control plants were watered every 5 days. After 12 days drought and normal condition treatments, the soil moisture content was measured using the method of weighing the soil before and after drying treatment. The soil moisture content under drought stress was reduced to around 17.29% from 49.86% under normal condition (Supplementary Figure S3). Tissues were collected 12 days after treatment. The shoots and roots were sampled separately and then stored at –80°C for RNA isolation. Three biological replicates were collected for each sample.

### Identification of Mungbean *VrDIR* Genes

The amino acid sequences of 25 *Arabidopsis* and 54 soybean DIR proteins were used as blast queries against the National Center for Biotechnology Information (NCBI) database to search for mungbean *VrDIR* candidate genes. All the output genes were analyzed using HMMER to confirm the conserved PF03018 domain (DIR domain) (Simon et al., 2018), and candidate genes containing conserved DIR domains were designated as *VrDIR* members. The gene ID, genomic length, and amino acid number were obtained from NCBI and mungbean database (https://legumeinfo.org/organism/Vigna/radiata) (http://plantgenomics.snu.ac.kr/mediawiki-1.21.3/index.php/Main_Page). ProtParam software (https://web.expasy.org/protparam/) was used to analyze the molecular weight (MW) of proteins and the theoretical iso-electric point (pI). The chromosome position of each *VrDIR* gene was obtained from NCBI and visualized using MapChart software (Voorrips, 2002).

### Phylogenetic Analysis

The amino acid sequences of DIR proteins from mungbean and other species reported by Corbin et al. (Corbin et al., 2018), including *DIR* members from *Arabidopsis thaliana*, *Forsythia x intermedia*, *Gossypium barbadense*, *Glycyrrhiza echinata*, *Gossypium hirsutum*, *Hordeum vulgare*, *Oryza sativa*, *Podophyllum peltatum*, *Picea sitchensis*, *Sorghum bicolor*, *Schizandra chinensis*, *Saccharum* hybrid cultivar, *Sesamum indicum*, *Triticum aestivum*, *Tamarix androssowii*, *Tsuga heterophylla*, *Thuja plicata*, *Linum usitatissimum*, *Zea mays*, *Glycine max*, *Vigna unguiculata*, *Pisum sativum* and *Phaseolus vulgaris* were aligned using ClustalW2 (Oliver et al., 2005). The phylogenetic tree was constructed using the alignment results of DIR proteins from these species in MEGA 7.0 using the neighbor-joining method with default parameters (Kumar et al., 2016). The classification of mungbean *VrDIR* genes was carried out as described by Corbin et al. (Corbin et al., 2018).

### Gene Duplication Analysis

Gene duplication of *VrDIR* genes was analyzed using OrthoMCL and Circos software following the methods of Jin et al. (Krzywinski et al., 2009; Fischer et al., 2011; Jin et al., 2019; Jin et al., 2020). Amino acid sequences with a similarity greater than 80% were designated as duplicated gene pairs.

### Exon-Intron Organization and Conserved Motif Analyses

The full lengths of each *VrDIR* genomic sequence and coding sequence were aligned using the Gene Structure Display Server program to analyze the exon-intron organization (Hu et al., 2015), and the UTRs, exons, and introns were shown in different colored boxes. The MEME tool was used to analyze VrDIR-conserved motifs with default parameters (Bailey et al., 2009), and the different motifs were displayed using different colored boxes.

### *Cis*-Acting Element Analysis of *VrDIR* Promoter Regions

The PlantCARE database (http://bioinformatics.psb.ugent.be/webtools/plantcare/html/) was used to predict the *cis*-acting elements in *VrDIR* promoter regions (Lescot et al., 2002). The promoter regions were analyzed using sequences 2 kb upstream of the initiation codon ATG. The *cis*-acting elements were classified into different groups based on their potential functions.

### RNA Isolation, cDNA Synthesis, and Gene Expression Analysis of *VrDIR* Genes

For RNA isolation and cDNA synthesis, the tissues were prepared as previously described (Li et al., 2021). Gene expression levels were analyzed using quantitative real-time PCR (qRT-PCR) as described by Ma et al. (Ma et al., 2021). All samples were analyzed using three biological replicates. The gene expression levels were normalized to a mungbean *Actin* gene (Li et al., 2018). All primers used in this study are listed in Supplementary Table S1.

## Results

### Identification of Mungbean *VrDIR* Genes

To search for mungbean *VrDIR* genes, we used the amino acid sequences of 25 *Arabidopsis* and 54 soybean DIRs as blast queries against the NCBI database. All of the output genes were analyzed using HMMER to confirm the conserved DIR domains (Simon et al., 2018). A total of 37 *VrDIR* genes were found in mungbean using genome-wide identification (Table 1). The genomic length of *VrDIRs* ranged from 444 (*XP_014,492,984*) to 4,131 bp (*XP_022,638,559*), and twenty-six *VrDIR* genes had a genomic length less than 1,000 bp. The CDS length ranged from 390 (*XP_014,499,484*) to 1,191 bp (*XP_014,497,369*), and the amino acid number varied from 129 to 396. Moreover, 28 out of the 37 VrDIR proteins had less than 200 amino acids, indicating that most VrDIRs are small proteins. The molecular weight of VrDIR proteins apparently ranged from 13,839.04 (XP_014,499,484) to 41,056.7 Da (XP_014,497,369), and the theoretical pI ranged from 4.42 (XP_014,497,369) to 9.55 (XP_014,493,094). In addition, 15 VrDIR proteins were predicted to be alkaline proteins (pI > 7.0), and 22 were predicted to be acidic proteins (pI < 7.0) (Table 1).

**TABLE 1:** *VrDIR* genes identified in mungbean.

Gene name	Gene ID (NCBI)	Gene ID	Chr	Genomic length/bp	CDS length/bp	AA length	MW/Da	pI	Sub-family
*VrDIR1*	XP_014,492,984	*Vradi01g01210*	1	444	444	147	16,249.55	8.04	e
*VrDIR2*	XP_014,493,926	*Vradi01g02190*	1	522	522	173	18,776.6	6.17	e
*VrDIR3*	XP_014,499,484	Unknown	1	2,799	390	129	13,839.04	4.95	e
*VrDIR4*	XP_014,493,581	Unknown	2	1,103	921	306	32,326.58	5.12	e
*VrDIR5*	XP_014,522,183	Unknown	2	966	753	250	27,167.92	6.23	e
*VrDIR6*	XP_014,523,732	*Vradi02g07650*	2	798	519	172	18,618.19	6.82	f
*VrDIR7*	XP_014,523,741	*Vradi02g07660*	2	600	546	181	19,635.36	9.51	f
*VrDIR8*	XP_014,523,749	Unknown	2	522	522	173	18,901.61	6.72	f
*VrDIR9*	XP_014,495,425	Unknown	3	828	579	192	20,806.77	9.36	b
*VrDIR10*	XP_014,494,945	Unknown	3	804	579	192	20,899	9.3	b
*VrDIR11*	XP_014,495,436	Unknown	3	836	576	191	20,594.58	8.99	b
*VrDIR12*	XP_014,495,544	Unknown	3	985	561	186	20,237.31	5.42	b
*VrDIR13*	XP_014,495,568	Unknown	3	1,276	561	186	20,238.29	5.22	b
*VrDIR14*	XP_014,495,583	Unknown	3	1,085	561	186	20,238.29	5.22	b
*VrDIR15*	XP_022,634,673	Unknown	3	1,239	651	216	23,980.6	6.05	b
*VrDIR16*	XP_014,497,584	*Vradi04g05580*	4	750	675	224	24,107.49	5.78	e
*VrDIR17*	XP_014,497,369	*Vradi04g10160*	4	2,341	1,191	396	41,056.7	4.42	e
*VrDIR18*	XP_014,500,237	Unknown	5	905	609	202	22,512.56	5.85	d
*VrDIR19*	XP_014,500,338	Unknown	5	858	573	190	20,974.73	5.78	d
*VrDIR20*	XP_014,508,512	Unknown	7	823	549	182	20,389.24	6.04	a
*VrDIR21*	XP_014,508,278	Unknown	7	2,141	498	165	17,882.45	5.63	e
*VrDIR22*	XP_022,638,559	Unknown	7	4,131	555	184	19,720.68	9.33	e
*VrDIR23*	XP_014,508,461	Unknown	7	631	546	181	20,098.99	6.18	f
*VrDIR24*	XP_014,507,775	Unknown	7	871	570	189	21,268.32	8.55	a
*VrDIR25*	XP_014,511,365	*Vradi08g01660*	8	649	567	188	20,342.31	8.96	b
*VrDIR26*	XP_014,513,034	Unknown	8	697	573	190	20,689.7	6.64	b
*VrDIR27*	XP_014,513,086	Unknown	8	1,162	738	245	25,066.62	4.87	e
*VrDIR28*	XP_014,512,994	Unknown	8	1,348	798	265	27,357.19	5.38	e
*VrDIR29*	XP_014,492,431	Unknown	Unknown	828	567	188	20,440.19	6.05	b
*VrDIR30*	XP_014,492,471	Unknown	Unknown	752	573	190	21,899.94	8	b
*VrDIR31*	XP_014,492,695	*Vradi0261s00120*	Unknown	982	576	191	21,304.56	8.95	b
*VrDIR32*	XP_014,492,698	Unknown	Unknown	1,031	570	189	21,071.15	9.25	b
*VrDIR33*	XP_014,492,697	*Vradi0261s00070*	Unknown	795	597	198	21,971.14	9.28	b
*VrDIR34*	XP_014,492,693	Unknown	Unknown	885	663	220	24,245.76	8.89	b
*VrDIR35*	XP_014,492,822	*Vradi0460s00010*	Unknown	925	564	187	20,474.31	6.51	b
*VrDIR36*	XP_014,493,094	*Vradi0410s00010*	Unknown	840	564	187	20,501.52	9.55	b
*VrDIR37*	XP_014,493,095	Unknown	Unknown	946	564	187	20,458.37	9.1	b

Chr, chromosome number; AA, amino acid; MW, molecular weight; pI, isoelectric point. Gene ID was obtained from mungbean genome database (https://legumeinfo.org/organism/Vigna/radiata) (http://plantgenomics.snu.ac.kr/mediawiki1.21.3/index.php/Main_Page).

The distribution of *VrDIRs* was uneven among the 11 mungbean chromosomes. Seven of the 11 chromosomes contained *VrDIR* genes, with the exception of chromosomes 6, 9, 10, and 11 (Table 1, Supplementary Figure S1). *VrDIR* genes were renamed from *VrDIR1* to *VrDIR28* based on their chromosome locations, and the other nine *VrDIR* genes were randomly designated from *VrDIR29* to *VrDIR37*. Among these chromosomes, chromosome three contained the most *VrDIR* genes, with seven members, followed by chromosomes 2 and 7, with five *VrDIRs* on each. Several *VrDIR* genes were located close to each other on the same chromosomes, such as *VrDIR4* and *VrDIR5*.

### Phylogenetic Relationships Among *VrDIR* Genes

**FIGURE 1:**
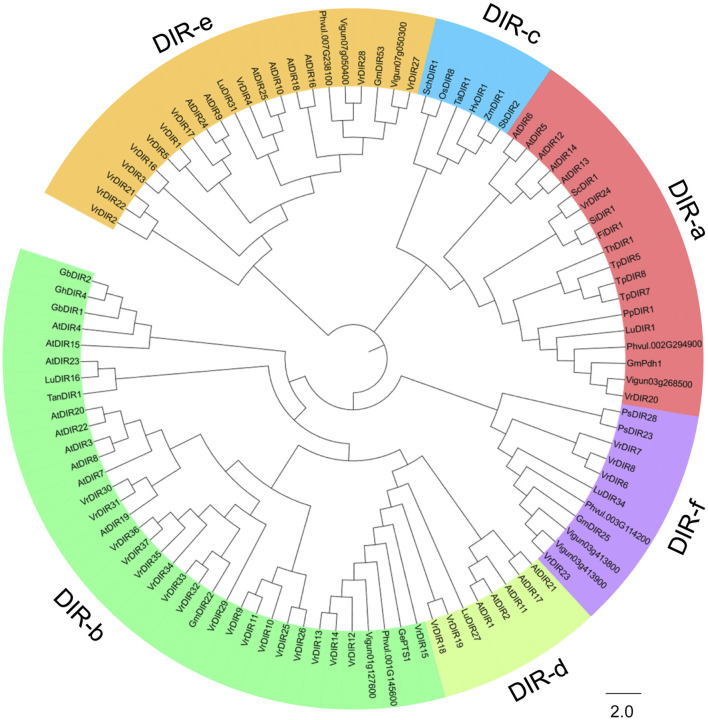
Analysis of the evolutionary relationships of DIR proteins from mungbean and other species. The amino acid sequences of DIR proteins from mungbean, *Arabidopsis*, and other species reported by Corbin et al. (2018) were used to conduct the phylogenetic analysis in MEGA 7.0 using the neighbor-joining method. DIR proteins were classified into six groups based on their phylogenetic relationships, DIR-a to DIR-f, which are indicated by different colors in the phylogenetic tree.

**FIGURE 2:**
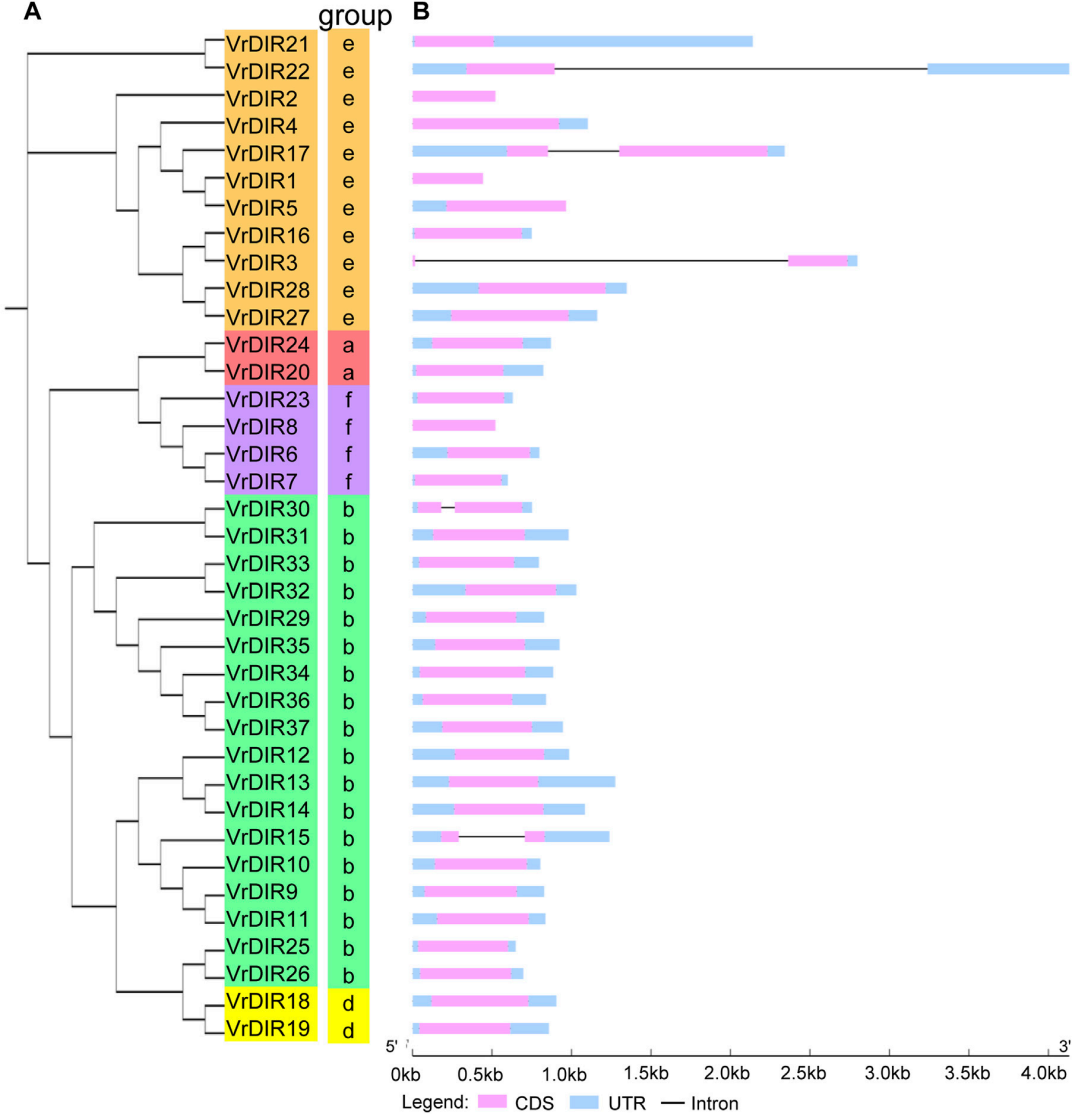
Phylogenetic and gene structure analyses of VrDIR genes. **(A)** Phylogenetic analysis of VrDIR proteins. The phylogenetic tree was constructed in MEGA 7.0 using the neighbor-joining method. **(B)** Exon-intron organization of *VrDIR* genes. The light blue, pink, and black boxes indicate UTRs, exons, and introns, respectively. The genomic lengths of *VrDIR* genes are indicated.

We constructed a phylogenetic tree of *DIR* genes from mungbean, soybean, common bean, cowpea, *Arabidopsis*, and other species examined by Corbin et al. to investigate the evolutionary relationships between *VrDIR* genes and *DIRs* in other species (Corbin et al., 2018). *VrDIR* genes were classified into five subclades based on their phylogenetic relationships (Figure 1). DIR-a, DIR-b, DIR-d, DIR-e, and DIR-f contained 2 (*VrDIR20*, *VrDIR24*), 18 (*VrDIR9*, *VrDIR10*, *VrDIR11*, *VrDIR12*, *VrDIR13*, *VrDIR14*, *VrDIR15*, *VrDIR25*, *VrDIR26*, *VrDIR29*, *VrDIR30*, *VrDIR31*, *VrDIR32*, *VrDIR33*, *VrDIR34*, *VrDIR35*, *VrDIR36*, *VrDIR37*), 2 (*VrDIR18*, *VrDIR19*), 11 (*VrDIR1*, *VrDIR2*, *VrDIR3*, *VrDIR4*, *VrDIR5*, *VrDIR16*, *VrDIR17*, *VrDIR21*, *VrDIR22*, *VrDIR27*, *VrDIR28*), and 4 (*VrDIR6*, *VrDIR7*, *VrDIR8*, *VrDIR23*) members, respectively (Figure 1). The homologous genes might have similar functions in different species, and we obtained some information for *VrDIRs* from the well-studied *DIRs* in other species based on their evolutionary relationships. *VrDIR30* and *VrDIR31* were closely related to *AtDIR19*, the expression level of which changed in response to heat stress (Paniagua et al., 2017), indicating that these two *DIR* genes might be involved in heat stress responses in mungbean. Moreover, *AtDIR9*, which is involved in the salt stress response, was classified into the same subgroup as *VrDIR17*, suggesting that *VrDIR17* is potentially involved in the salt stress response (Figure 1). We also constructed a phylogenetic tree using only *VrDIR* genes and found that *VrDIR* genes in each group were clustered together in the phylogenetic tree (Figure 2).

### Gene Structure and Conserved Motif Analyses of *VrDIR* Genes

**FIGURE 3:**
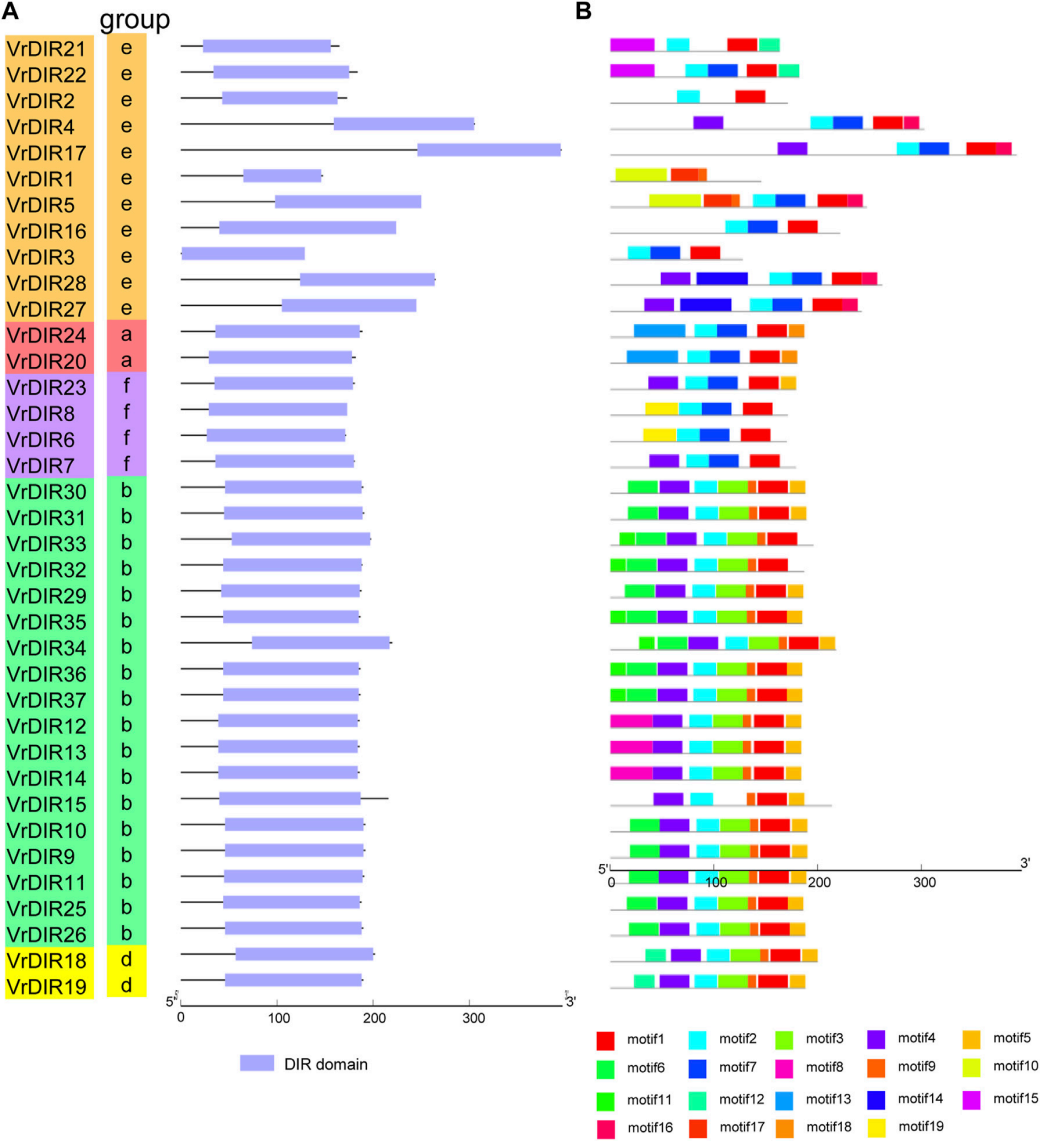
Conserved domain and motif analyses in VrDIR proteins. **(A)** The positions and lengths of the conserved DIR domains in VrDIR proteins. The light blue boxes indicate the conserved DIR domains. **(B)** The conserved motifs in VrDIR proteins. The 19 motifs are indicated using different colored boxes. The length of each DIR protein is indicated.

To investigate the exon-intron organization of *VrDIR* genes, we used the Gene Structure Display Server program to analyze *VrDIR* genomic and coding sequences. Thirty-two out of the 37 *VrDIRs* had a classical *DIR* gene structure, one exon without introns; the exceptions were *VrDIR3*, *VrDIR15*, *VrDIR17*, *VrDIR22*, and *VrDIR30*, which each had one intron (Figure 2). Moreover, most of the *VrDIR* genes contained UTRs, with the exception of *VrDIR1*, *VrDIR2*, and *VrDIR8*, which had only one exon (Figure 2). The conserved DIR domain occupied the majority of the protein in most VrDIRs, with the exception of DIR-e subfamily members VrDIR4, VrDIR17, VrDIR27, and VrDIR28 (Figure 3). Next, we analyzed the conserved motifs of VrDIR proteins using MEME tools; a total of 19 distinct motifs were detected in all 37 VrDIR proteins (Figure 3, Supplementary Figure S2). Motif one was present in all VrDIR proteins, which might indicate the conserved DIR domain (Figure 3). The differences in the motifs reflect the diversity of VrDIR proteins. For example, motifs three and nine occurred in the DIR-b and DIR-d groups, and motif 19 was only present in two DIR-f genes, which indicates that VrDIR proteins are functionally diverse (Figure 3).

### Duplication Analysis of *VrDIRs*

**FIGURE 4:**
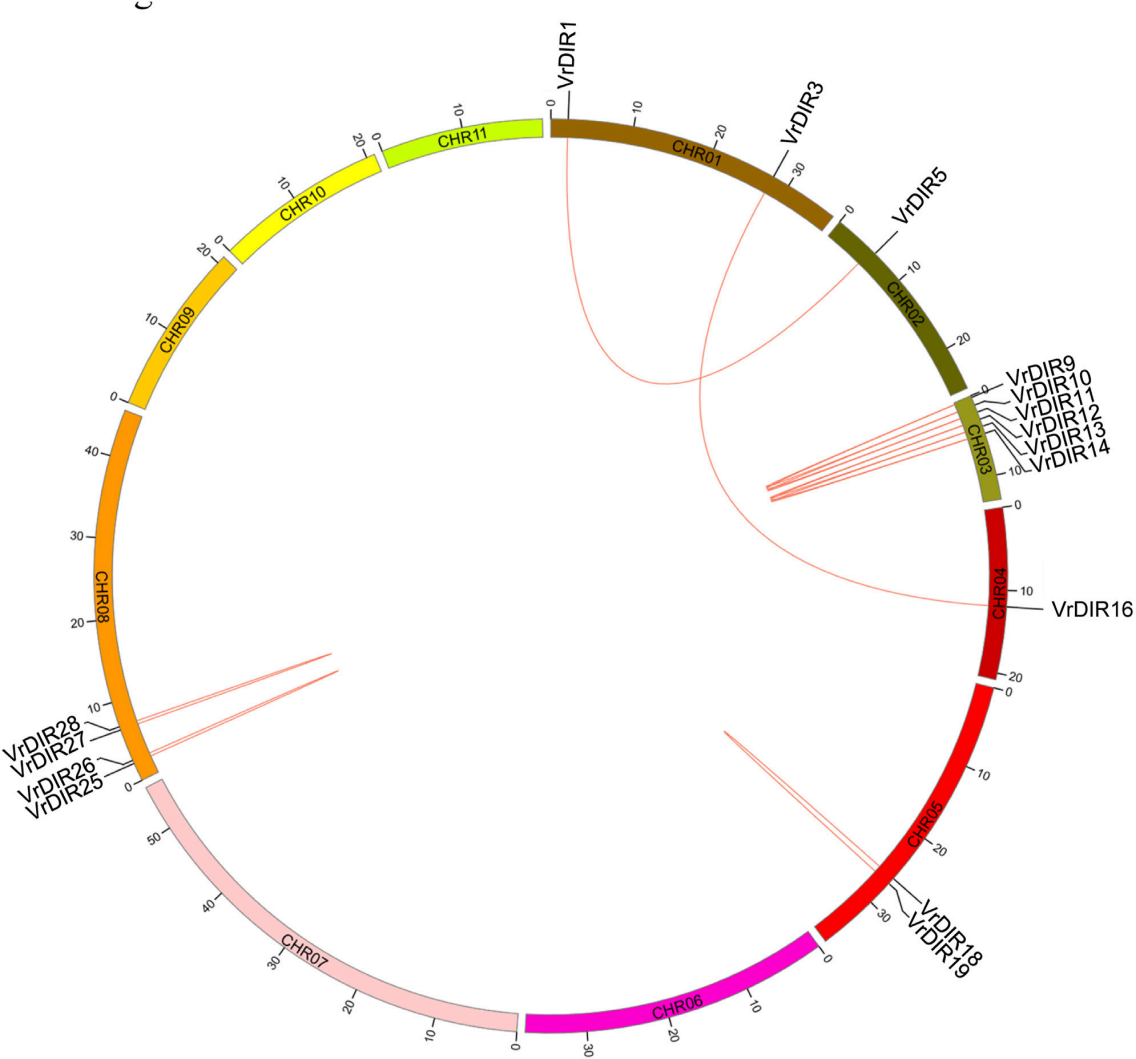
Duplication analysis of VrDIR genes. The positions of *VrDIR* genes in each chromosome are presented, and the duplicated gene pairs are connected using red lines. The length of each chromosome is indicated, and CHR indicates chromosome.

Duplicated gene pairs are produced during whole-genome duplication in plants (Li et al., 2019; Ma et al., 2021). Duplication events were analyzed among *VrDIR* members. A total of 13 duplicated gene pairs were observed among 37 *VrDIR* genes, which is consistent with the high similarity observed in mungbean *DIR* genes. Eleven duplicated gene pairs are shown in Figure 4, including *VrDIR1/VrDIR5*, *VrDIR3/VrDIR16*, *VrDIR9/VrDIR10*, *VrDIR9/VrDIR11*, *VrDIR10/VrDIR11*, *VrDIR12/VrDIR13*, *VrDIR12/VrDIR14*, *VrDIR13/VrDIR14*, *VrDIR18/VrDIR19*, *VrDIR25/VrDIR26*, and *VrDIR27/VrDIR28*; the duplicated gene pairs *VrDIR36/VrDIR37* and *VrDIR32/VrDIR33* were discarded because of a lack of chromosome information. The duplicated genes were clustered into a clade in the phylogenetic tree (Figure 2). Chromosome three contained the most duplicated genes, including *VrDIR9*, *VrDIR10*, *VrDIR11*, *VrDIR12*, *VrDIR13*, and *VrDIR14*; chromosomes 1, 2, 4, 5, and eight contained 2, 1, 1, 2, and four duplicated genes, respectively (Figure 4). *VrDIR10/VrDIR11*, *VrDIR12/VrDIR13*, *VrDIR13/VrDIR14*, *VrDIR18/VrDIR19*, and *VrDIR27/VrDIR28* were derived from tandem duplication events, whereas *VrDIR1/VrDIR5* and *VrDIR3/VrDIR16* represented interchromosomal duplicated gene pairs (Figure 4).

### *Cis*-Acting Element Analysis of *VrDIR* Promoters

**FIGURE 5:**
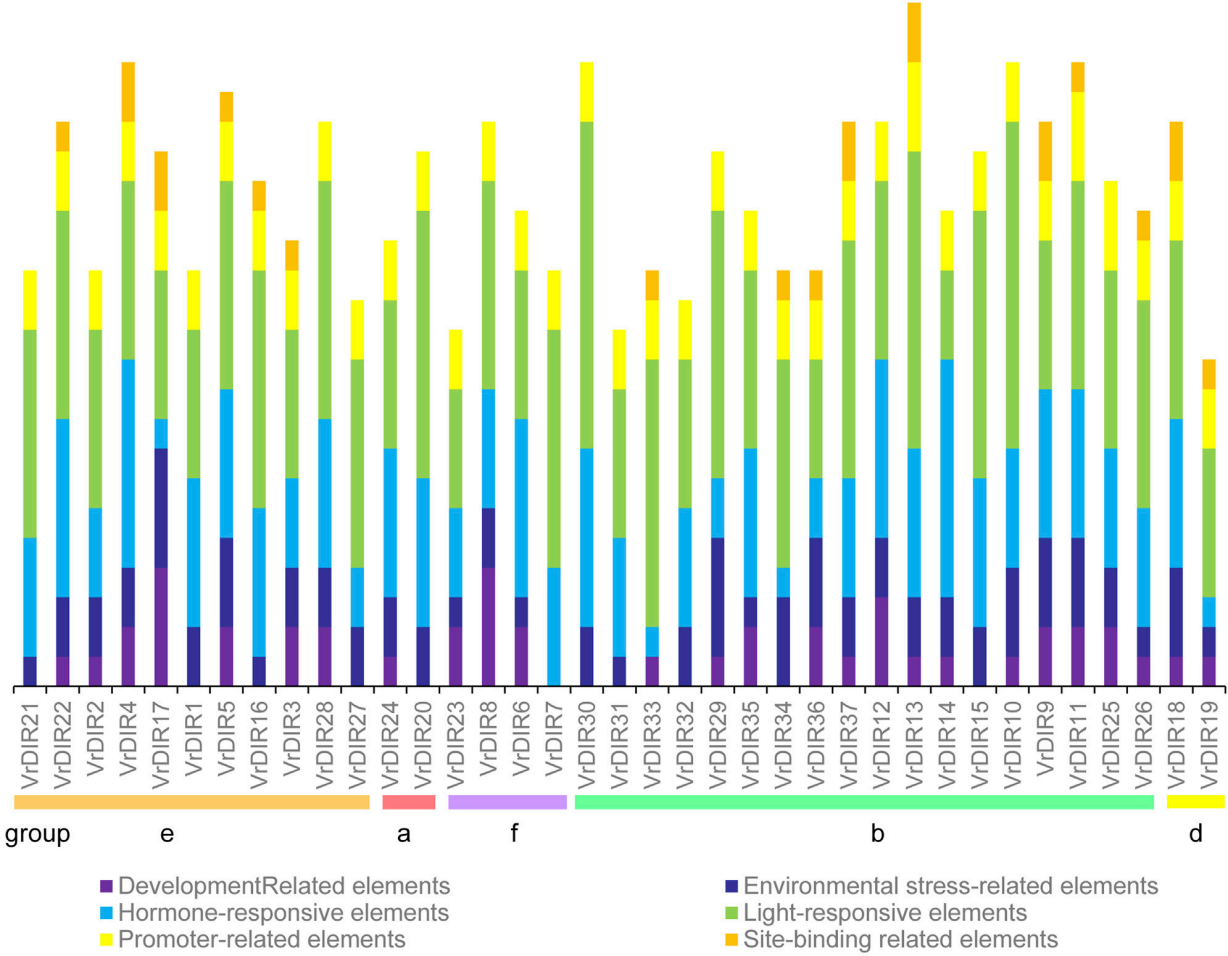
*Cis*-acting element analysis in *VrDIR* promoter regions. The *cis*-acting elements were classified into six groups based on their predicted functions as described by Jin et al. (2019). Different *cis*-acting elements were presented using different colored boxes, and the Y-axis indicates the number of *cis*-acting elements in *VrDIR* promoters.

*Cis*-acting elements mediate various responses by regulating promoter activities; we predicted *cis*-acting elements in *VrDIR* promoter regions using sequences 2 kb upstream of each initiation codon. A total of 92 *cis*-acting elements were found in all 37 *VrDIR* promoter regions, and 57 *cis*-acting elements had predicted functions (Supplementary Table S2), which were classified into six different groups according to their potential functions (Figure 5) (Ma et al., 2021). Light-responsive elements were the most abundant in 34 *VrDIR* genes; in the promoter regions of *VrDIR4*, *VrDIR6*, and *VrDIR14*, hormone-responsive elements were the most abundant, indicating that these three *VrDIRs* are involved in hormone responses (Figure 5). Moreover, the DIR-b, DIR-d, and DIR-e subfamilies contained all six groups of *cis*-acting elements, and DIR-a and DIR-f subfamilies contained only five groups of *cis*-acting elements, with the exception of ‘site-binding related elements’ (Figure 5), which suggests that the expression of these *VrDIR* genes in response to stress varies. In addition, the *cis*-acting elements in duplication events differed in some gene pairs, indicating that they might have different functions (Figure 5). For example, the duplicated genes *VrDIR13* and *VrDIR14* have different numbers of hormone-responsive elements, light-responsive elements, promoter-related elements, and site-binding-related elements, suggesting that these two genes might show different responses to stress (Figure 5, Supplementary Table S2).

### Analysis of *VrDIR* Expression in Different Tissues

**FIGURE 6:**
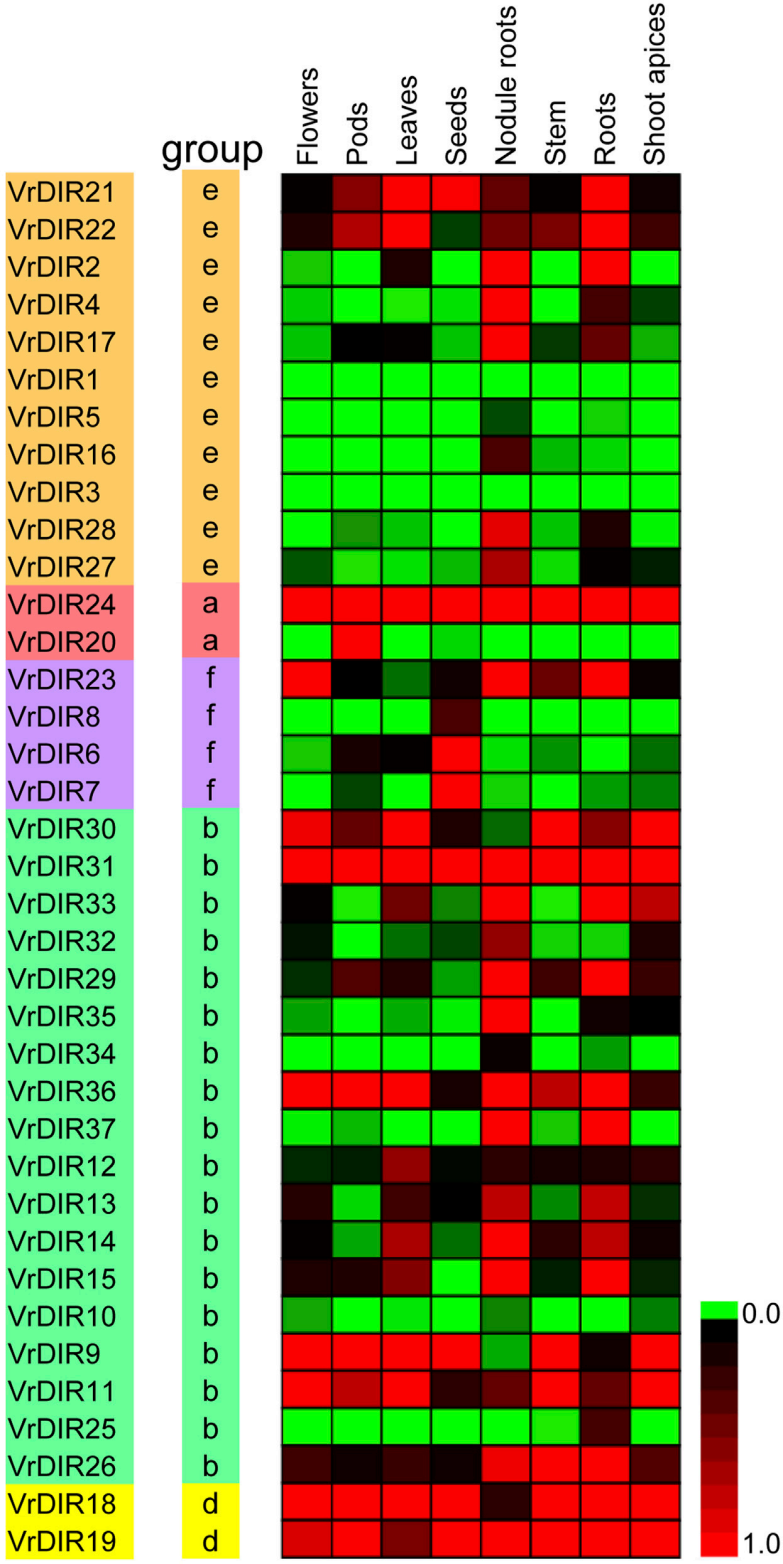
Relative expression levels of *VrDIR* genes in different tissues. Eight tissues, including flowers, pods, leaves, seeds, nodule roots, stems, roots, and shoot apices, were used for analysis. The expression level of *VrDIR37* in nodule roots was set as one, and the others were adjusted accordingly. The gene expression results were visualized using a heatmap generated with Multiple Experiment Viewer 4.9.0 (Saeed et al., 2003). The expression levels from 0 to one are indicated by different colors.

We analyzed the expression levels of *VrDIRs* in eight different tissues: flowers, pods, leaves, seeds, nodule roots, stems, roots, and shoot apices (Figure 6). The expression levels of *VrDIRs* varied among tissues. For example, the expression of *VrDIR1* and *VrDIR3* was low in all tested tissues, indicating that these two genes might have weak functions in these tissues. In contrast, the expression of *VrDIR24* and *VrDIR31* was high in all tissues (Figure 6), indicating that *VrDIR24* and *VrDIR31* have important functions in these tissues. In addition, duplicated genes might retain some common functions as well as evolve new functions. Thus, several duplicated genes showed similar expression levels in some tissues, and expression patterns differed in other tissues (Figure 6). For example, *VrDIR9* and *VrDIR11* showed similar expression patterns in flowers, leaves, stems, and shoot apices, but different expression levels in pods, seeds, nodule roots, and roots, indicating that *VrDIR9* and *VrDIR11* might have similar functions in some tissues but different roles in other tissues.

### Expression of *VrDIR* Genes in Response to Salt and Drought Stress

**FIGURE 7:**
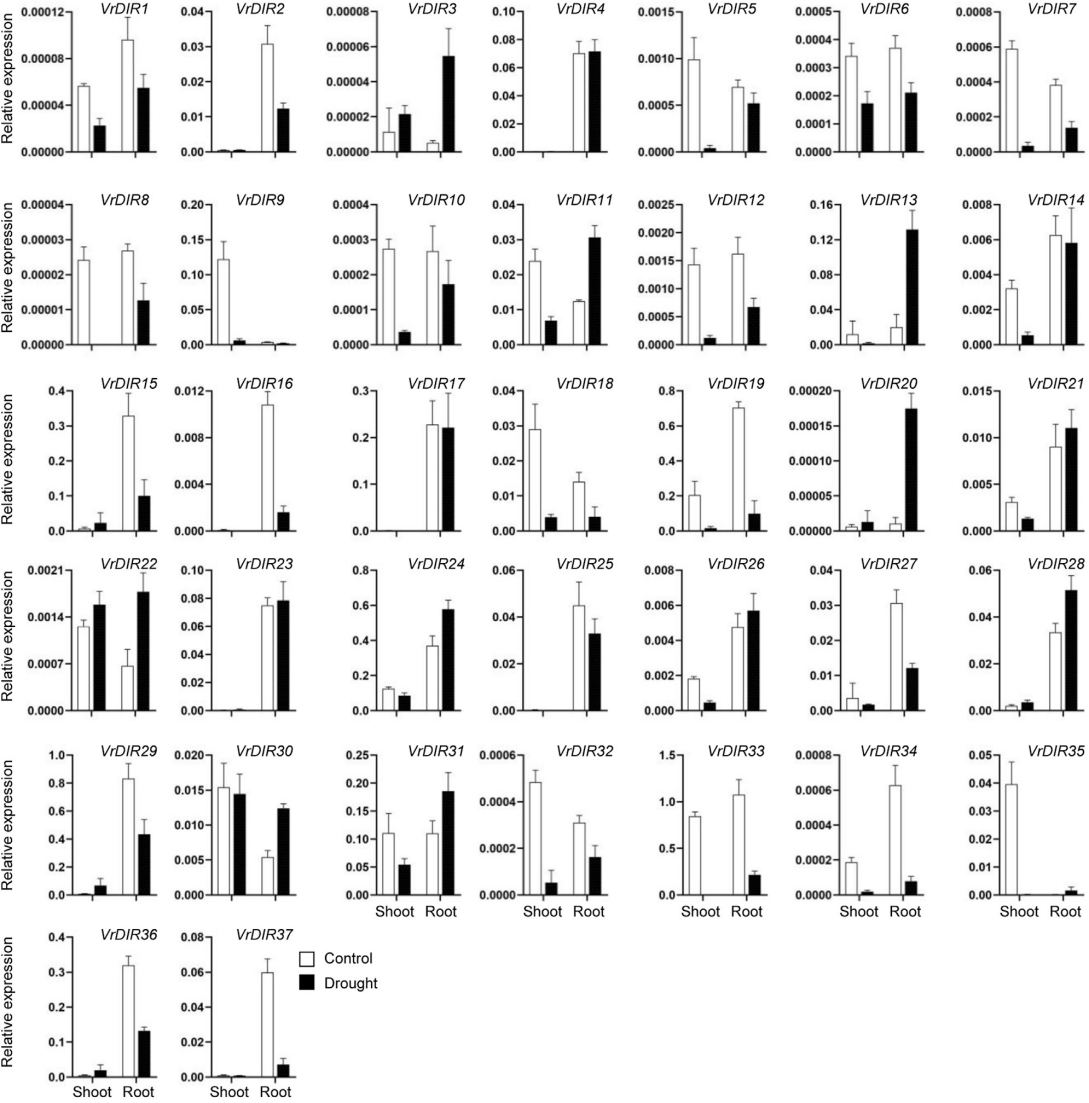
Expression levels of *VrDIR* genes in response to drought stress. The expression of *VrDIR* genes in shoots and roots grown under normal and drought conditions was analyzed using qRT-PCR. Each sample was analyzed using three biological replicates and normalized to an *Actin*-expressing gene in mungbean.

**FIGURE 8:**
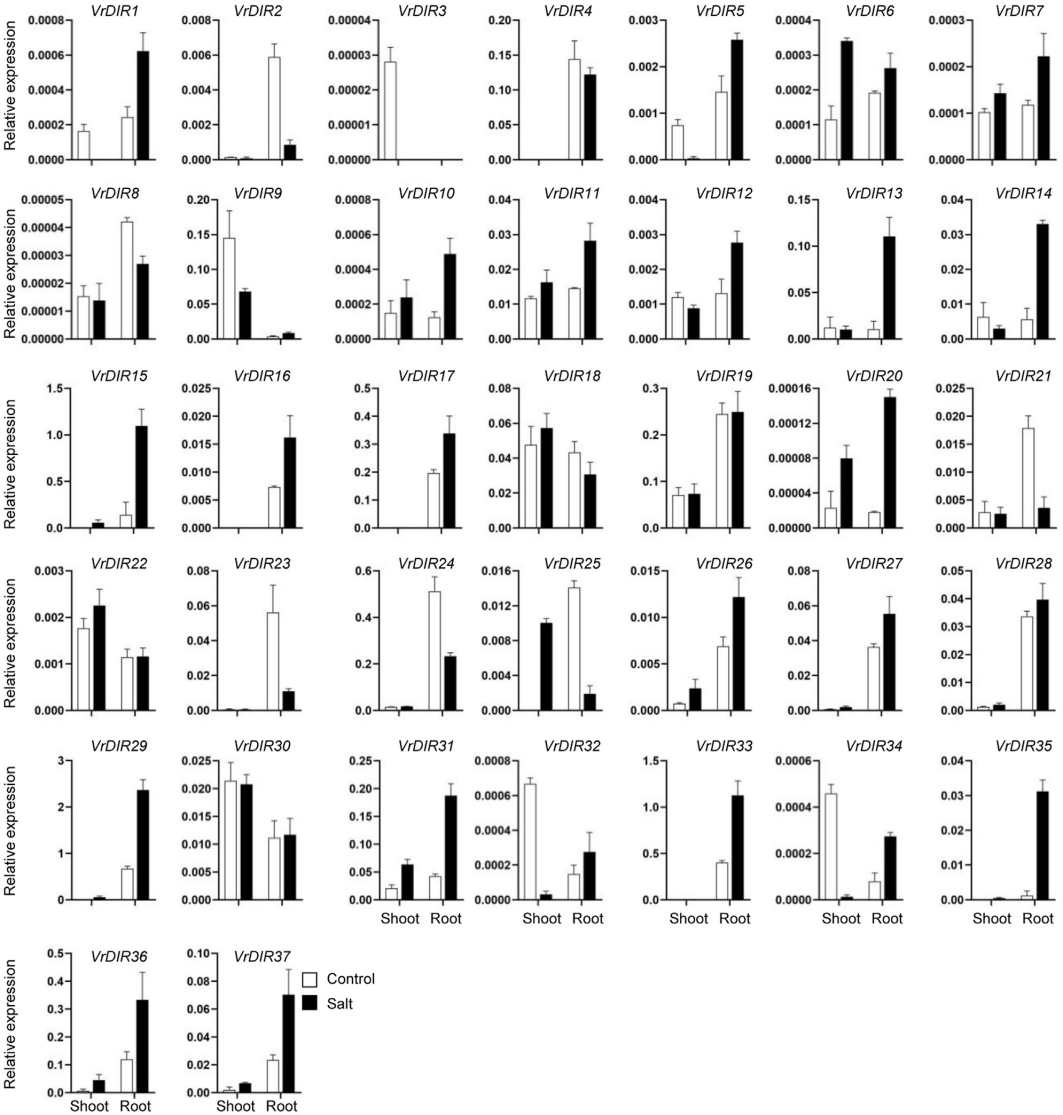
Expression levels of *VrDIR* genes in response to salt stress. The expression of *VrDIR* genes in shoots and roots grown under normal and salt conditions was analyzed using qRT-PCR. Each sample was analyzed using three biological replicates and normalized to an *Actin*-expressing gene in mungbean.

Next, we analyzed the expression of *VrDIR* genes in mungbean shoots and roots under salt and drought stress. The fresh weights of mungbean plants were significantly lower under salt or drought treatment than that under normal conditions which indicated that the growth of mungbean plants was inhibited after salt or drought stress treatment (Supplementary Figure S3). *VrDIR* genes varied in their responses under different stress conditions (Figures 7, 8). Most of the *VrDIR* genes showed altered expression levels under both drought and salt stress conditions in shoots and roots, with the exception of *VrDIR4*, whose expression was not affected by salt or drought stress (Figures 7 and 8). For example, the expression level of *VrDIR34* decreased in both shoots and roots under drought stress and increased in roots and decreased in shoots under salt stress (Figures 7 and 8). Some *VrDIR* genes only exhibited responses to either drought or salt stress. For example, the expression of *VrDIR19* decreased in both shoots and roots under drought conditions; however, the expression of *VrDIR19* was not affected by salt stress (Figures 7 and 8). Moreover, the expression of *VrDIR25* in different tissues did not vary under drought stress but increased and decreased in shoots and roots under salt stress, respectively (Figures 7 and 8), indicating that this *VrDIR* gene might have different functions in response to drought and salt stress.

## Discussion

In previous decades, several studies have characterized *DIR* genes, and this work has greatly increased our understanding of the responses of plants to stress in many species. Many *DIR* gene expression studies correlated their involvement in biotic and abiotic stress. In this study, we characterized 37 *DIR* genes from mungbean and analyzed their expression patterns in response to drought or salt stress.

The typical structure of a *DIR* gene contains one exon and no introns (Corbin et al., 2018). In mungbean, 32 *VrDIR* genes contained this typical gene structure, and five *VrDIR* genes, including *VrDIR3*, *VrDIR15*, *VrDIR17*, *VrDIR22*, and *VrDIR30* (all of which belong to groups DIR-b and DIR-e), contained introns. In contrast, all DIR-a, DIR-d, and DIR-f members contained the typical gene structure (Figure 2), indicating that the gene structure of *VrDIR* genes is conserved in these three subfamilies. In soybean, eight *DIR* genes contain introns, seven of which belong to groups DIR-b and DIR-e (Ma et al., 2021). The conserved DIR domain occupies the majority of typical DIR proteins (Corbin et al., 2018), and the DIR domain in several mungbean DIRs, including VrDIR4, VrDIR17, VrDIR27, and VrDIR28, made up less than half of the protein (Figure 3), all of which belonged to group DIR-e. These results indicated that the gene structure differed in DIR-b and DIR-e members; other group members show high conservation in their sequences. In addition, some motifs, such as motif 1, were observed in all VrDIR proteins, indicating the conservation of *VrDIR* genes. However, some motifs are only present in specific subfamilies, such as motif three and motif 9, which reflects the high functional diversity of VrDIR proteins in different subfamilies (Figure 3).

The legume plants were considered to be evolved from the same origin million years ago, and the genomes of different species have been modified in various ways over evolutionary time (Schmutz et al., 2010; Young et al., 2010; Kang et al., 2014). Mungbean and *Medicago* underwent one round of whole-genome duplication. Mungbean contained 37 *DIRs*, and *Medicago* plants contained 45 *DIRs* (Table 1) (Young et al., 2010; Song and Peng, 2019), indicating that some *DIR* genes might have been lost in mungbean during their evolutionary history. Soybean has undergone two rounds of whole-genome duplication and had 54 *GmDIR* genes, which is less than twice the number in mungbean or *Medicago* (Schmutz et al., 2010; Ma et al., 2021). These results suggest that the numbers of *DIR* genes have changed extensively during the evolutionary history of legumes. Moreover, *DIRs* were grouped into seven groups (DIR-a to DIR-g) in flax (*Linum usitatissimum* L.); they were only grouped into five groups in mungbean and did not have a DIR-c member, which only occurs in monocot species, nor a DIR-g member, which only occurs in flax (Corbin et al., 2018), indicating that these two groups were lost during mungbean evolution. Mungbean contained two DIR-d group members, *VrDIR18* and *VrDIR19* (Figure 1). However, soybean has no DIR-d members, indicating that the types of *DIR* genes changed during the evolution of legumes (Ma et al., 2021). In addition, the *VrDIR* genes in some subfamilies underwent several duplication events during evolution. For example, all of the duplicated gene pairs were present in the DIR-b, DIR-d, and DIR-e subfamilies, whereas no duplication events were observed in the DIR-a and DIR-f subfamilies (Figure 4), which reflects the evolutionary diversity of these subfamilies. Chromosome three contained the most duplicated genes, and most of the *VrDIR* genes located on chromosome three had duplicated genes, with the exception of *VrDIR15* (Figure 4), indicating that chromosome three might contain the original genes of many duplicated *VrDIR* genes (Li et al., 2019). *VrDIRs* in the same subfamily showed distinct expression patterns in different tissues, which suggests that these *VrDIRs* are functionally diverse. For example, the DIR-a subfamily members *VrDIR20* and *VrDIR24* are thought to be involved in pinoresinol formation (Ralph et al., 2006; Corbin et al., 2018). *VrDIR24* showed high expression levels in all tested tissues, whereas *VrDIR20* was only highly expressed in pods. The different *VrDIR* subfamilies contained different types of *cis*-acting elements. For example, DIR-b, DIR-d, and DIR-e subfamilies contained all six groups of *cis*-acting elements, whereas the DIR-a and DIR-f subfamilies contained only five groups, which might be responsible for the different expression patterns of *VrDIR* genes in different tissues (Figures 5, 6). Although *VrDIR1* and *VrDIR3* promoters contained many *cis*-acting elements, they showed extremely low expression levels in all tested tissues (Figure 6). The expression levels of genes are affected by many factors aside from *cis*-acting elements, such as temporal and spatial factors. *VrDIR1* and *VrDIR3* might be expressed at other time points in response to the environment. Duplicated genes might have the same origin and similar functions. Thus, *VrDIR9* and *VrDIR11* might retain similar functions in flowers, leaves, stems, and shoot apices and evolve new functions in pods, seeds, nodule roots, and roots based on their expression patterns (Figure 6). Moreover, mungbean had 13 *VrDIR* duplicated gene pairs, nearly half of that in soybean, which contained 24 *GmDIR* duplicated gene pairs (Ma et al., 2021), indicating that the evolution of duplicated gene pairs might be conserved in these two legumes. In *Arabidopsis*, 16 of the 25 *AtDIR* genes are highly expressed in the roots (Paniagua et al., 2017); in contrast, only a portion of *VrDIR* genes was highly expressed in mungbean roots (Figure 6), indicating that the functions of many *DIR* genes in *Arabidopsis* and mungbean have diverged. Moreover, homologous genes might have similar functions in different species. The expression of *AtDIR9* changed under salt stress, and the expression of its close homologs *VrDIR1*, *VrDIR5*, and *VrDIR17* increased under salt stress in roots; the expression of *VrDIR1* and *VrDIR5* in the shoots decreased. The expression of *AtDIR5* is altered under salt and drought stress (Paniagua et al., 2017), and the expression of its close homologs *VrDIR20* and *VrDIR24* was altered in the shoots and roots (Figures 7, 8). The expression of *AtDIR5* is altered in response to methyl jasmonate, wounding, and oxidative stress (Paniagua et al., 2017), suggesting that *VrDIR20* and *VrDIR24* might be involved in the regulation of these various types of stress in mungbean.

In sum, we identified and characterized 37 *VrDIR* genes in mungbean and characterized the phylogenetic relationships, exon-intron organization, conserved motifs, duplication events, *cis*-acting elements, and expression patterns in different tissues. We also analyzed the expression patterns of *VrDIR* genes in response to drought and salt stress. Our study provides basic information for future studies of *VrDIR* genes and their role in stress responses.

## Data Availability

The original contributions presented in the study are included in the article/Supplementary Material, further inquiries can be directed to the corresponding author.
