# Supplementary material for: Mungbean DIRIGENT Gene Subfamilies and Their Expression Profiles Under Salt and Drought Stresses
Source: Front Genet. 2021 Sep 22;12:658148. doi: 10.3389/fgene.2021.658148 (PMC8493098; doi:10.3389/fgene.2021.658148)
Supplement: Supplementary file 3 [file Table1.docx]

Supplementary Material

Mungbean *DIRIGENT* gene subfamilies and their expression profiles under salt and drought stresses

Wenying Xu^2, #^, Tong Liu^2, #^, Huiying Zhang^2, #^, Hong Zhu^1, *^

*Correspondence: Hong Zhu: zhuhong@qau.edu.cn


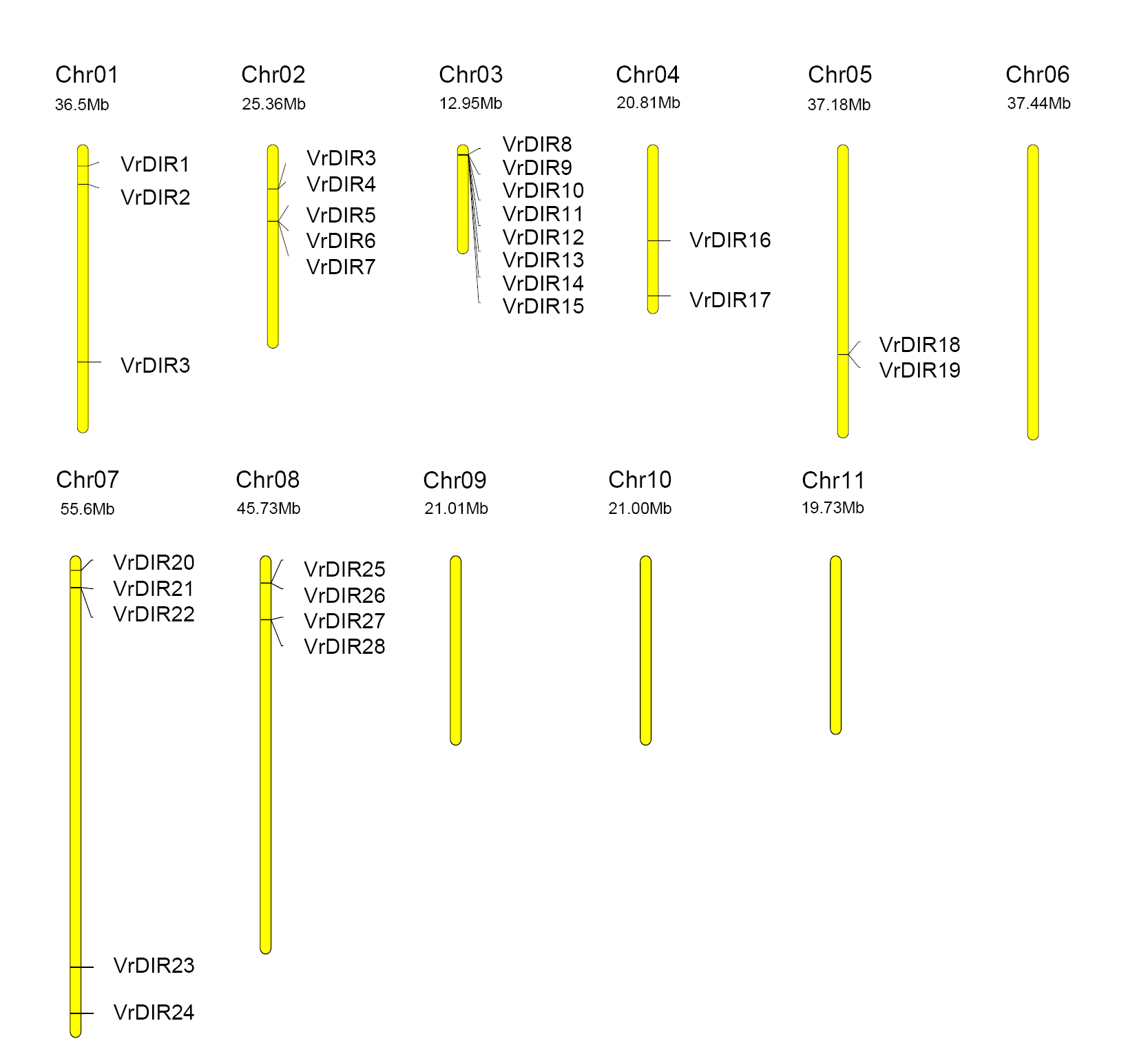


**Supplementary Figure S1 Chromosomal location of *VrDIR* genes.** The positions of *VrDIR* genes and chromosome lengths are indicated.


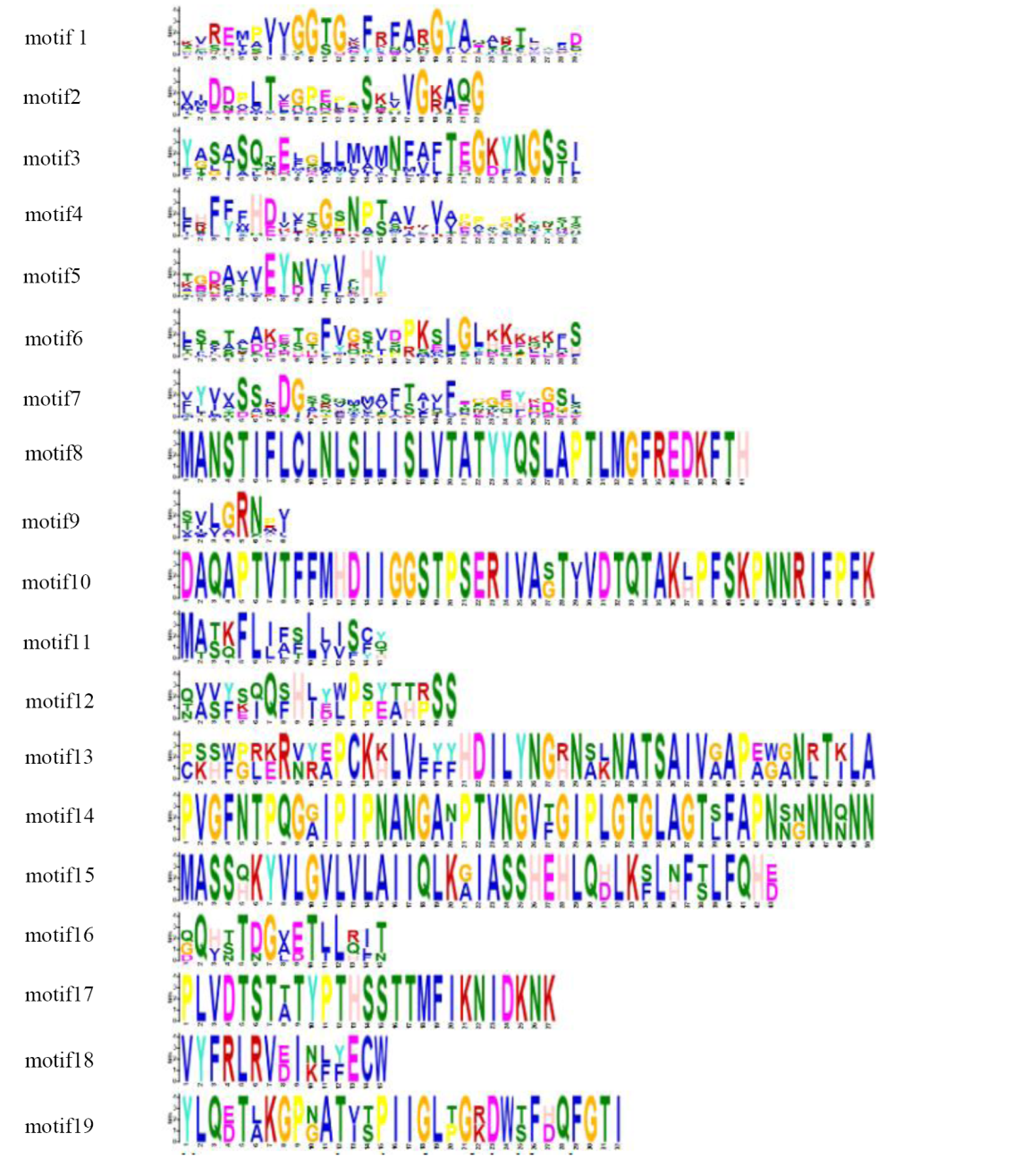


**Supplementary Figure S2 Sequence logos of 19 conserved motifs in VrDIR proteins.** The “sites” indicates the number of VrDIR proteins containing each motif, and the “width” indicates the amino acid number of each motif.


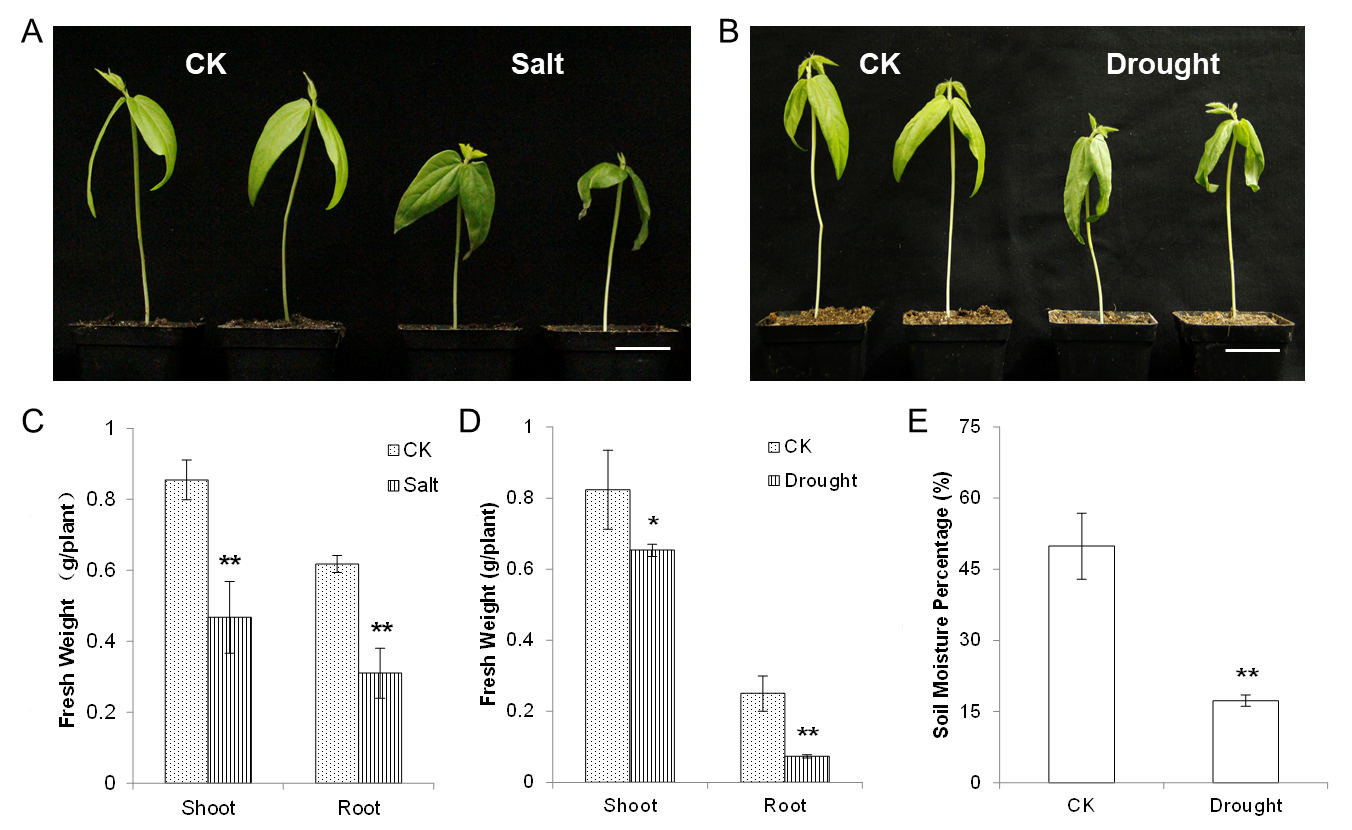


**Supplementary Figure S3 Growing condition and soil moisture percentage for control and stress treatments.** (A) Growing condition of *VC1973A* under control or salt stress condition. (B) Growing condition of *VC1973A* under control or drought stress condition. (C) Fresh weight of *VC1973A* under control or salt stress condition. (D) Fresh weight of *VC1973A* under control or drought stress condition. (E) Soil moisture percentage of *VC1973A* under control or drought stress condition.
